# The dissection of tomato flavor: biochemistry, genetics, and omics

**DOI:** 10.3389/fpls.2023.1144113

**Published:** 2023-06-06

**Authors:** Gurleen Kaur, Modesta Abugu, Denise Tieman

**Affiliations:** ^1^ Horticultural Sciences Department, University of Florida, Gainesville, FL, United States; ^2^ Department of Horticulture Science, North Carolina State University, Raleigh, NC, United States

**Keywords:** tomato flavor, aroma, volatiles, omics, machine learning, gene editing

## Abstract

Flavor and quality are the major drivers of fruit consumption in the US. However, the poor flavor of modern commercial tomato varieties is a major cause of consumer dissatisfaction. Studies in flavor research have informed the role of volatile organic compounds in improving overall liking and sweetness of tomatoes. These studies have utilized and applied the tools of molecular biology, genetics, biochemistry, omics, machine learning, and gene editing to elucidate the compounds and biochemical pathways essential for good tasting fruit. Here, we discuss the progress in identifying the biosynthetic pathways and chemical modifications of important tomato volatile compounds. We also summarize the advances in developing highly flavorful tomato varieties and future steps toward developing a “perfect tomato”.

## 1 Introduction

Consumer dissatisfaction with the flavor of many modern fruits and vegetables is well documented, but an understanding of the underlying biochemical and molecular bases for good flavor is necessary. Modern fresh market tomatoes with a round shape, large size, red color, and disease resistance proved not to be enough to attract consumers as they are tasteless (Tieman et al., 2017). Several factors have contributed to the loss of tomato flavor in modern tomato varieties. It is difficult to breed for tomato flavor because of genetic complexity, environmental effects, and expensive phenotyping. Additionally, the flavor components must be present in the fruit in quantities consistent with good flavor balance (Mathieu et al., 2009). Plant breeders have improved traits of great importance to producers, including yield, size, shape, and disease resistance of fresh market tomatoes. However, breeding for these traits resulted in an unintentional loss of tomato flavor (Folta and Klee, 2016). Linkage drag associated with quantitative traits such as fruit weight and disease resistance also contributed to the loss of positive contributors to flavor (Li et al., 2022). Additionally, growers are paid for the production quantity, not flavor quality, leading to limited interest in growing tomato varieties with good flavor, but lower yield (Klee and Tieman, 2018). There is some interest in producing a high-quality product among greenhouse and indoor agriculture farmers as consumers’ demand and willingness to pay for good flavor has increased (Figàs et al., 2015).

Tomato’s unique flavor results from a balance of sugars, acids, and volatile organic compounds (VOCs). The most common sugars in tomato fruits are glucose and fructose, and the most common acids are citrate, malate, and ascorbate. Over 400 VOCs have been detected in tomato with approximately 25–30 contributing significantly to the flavor of the fruit (Baldwin and Thompson, 2000; Klee and Tieman, 2013; Tieman et al., 2017; Klee and Tieman, 2018). Most of these VOCs are derived from essential nutrients such as carotenoids, essential amino acids, and essential fatty acids (Goff and Klee, 2006). Based on their biochemical origin, they are grouped as branched-chain amino acid (BCAA)-derived, phenylalanine-derived, carotenoid-derived (Apocarotenoids), and fatty acid-derived volatiles (Klee, 2010). While some of the pathways involved in their biosynthesis are known, many of the genes involved in the regulation of gene expression are still unknown. Identifying and validating the genes involved in VOCs biosynthesis and regulation is a major step towards the development of tomatoes with improved flavor.

Although increasing sugar content can improve flavor, altering sugar levels could affect fruit size as there is a negative correlation between size and sugar. However, VOCs can be detected in nanogram quantities by the human olfactory system, so their levels can be altered without affecting fruit size. Some VOCs are also found to enhance the perception of sweetness for consumers without altering sugar content (Tieman et al., 2012). For example, Colantonio et al. (2022) reported that 62% of tomato sweetness can be explained by VOCs, while sugars explained approximately 29% of tomato sweetness. Several studies combining genetic, sensory analysis, chemical, and metabolomic research have made it possible to predict the sweetness intensity and overall liking in tomatoes. Most of these studies recommend increasing the levels of some desirable VOCs, while decreasing the levels of undesirable compounds to significantly improve tomato flavor (Tieman et al., 2012; Tieman et al., 2017; Klee and Tieman, 2018; Gao et al., 2019; Zhao et al., 2019; Colantonio et al., 2022).

The development of tomato reference genomes has facilitated a better understanding of mechanisms involved in the biosynthesis of tomato VOCs. Similarly, utilization of genomes, transcriptomes, proteomes, and metabolomes has made identification of genetic variation, predictions of gene functions, posttranscriptional regulation, and modifications possible (Zhu et al., 2019). In this review, we summarize the pathways involved in the biosynthesis of tomato VOCs, biochemical modifications to volatiles that change their flavor attributes, and progress in flavor research using omics tools. Many VOCs including 3-methylbutanol, 4-methylpentanol, 3-methylpentanol, (*Z*)-3-hexen-1-ol, 6-methyl-5-hepten-2-ol, guaiacol, 2-phenylethanol, methyl salicylate, and eugenol are present in both free and glycosidically bound forms (Birtić et al., 2009). Although glycosides do not contribute to the fruit flavor directly, it is important to determine their role in VOC regulation as many glycosides can be cleaved to produce free volatiles without new biosynthesis of the volatiles (Liang et al., 2022). We also discuss recent advances and recommended next steps, towards developing a flavorful tomato.

## 2 Pathways for synthesis of tomato volatiles

Almost all important volatile compounds related to flavor are derived from essential nutrients such as carotenoids (lycopene and β-carotene), essential amino acids (phenylalanine, leucine, and isoleucine), and essential fatty acids (principally linoleic and linolenic acids) (Goff and Klee, 2006). While some of the pathways involved in tomato volatile biosynthesis have been identified, many steps in the biochemical pathways have not been defined. In addition, understanding of the enzymes and genes involved in their regulation continues to be an active area of research (Goulet et al., 2012; Mageroy et al., 2012; Shen et al., 2014; Goulet et al., 2015). Here, we summarize the current knowledge of biosynthetic pathways and genes involved in tomato volatile biosynthesis and regulation.

### 2.1 Branched-chain amino acid-derived volatiles

**Figure 1:**
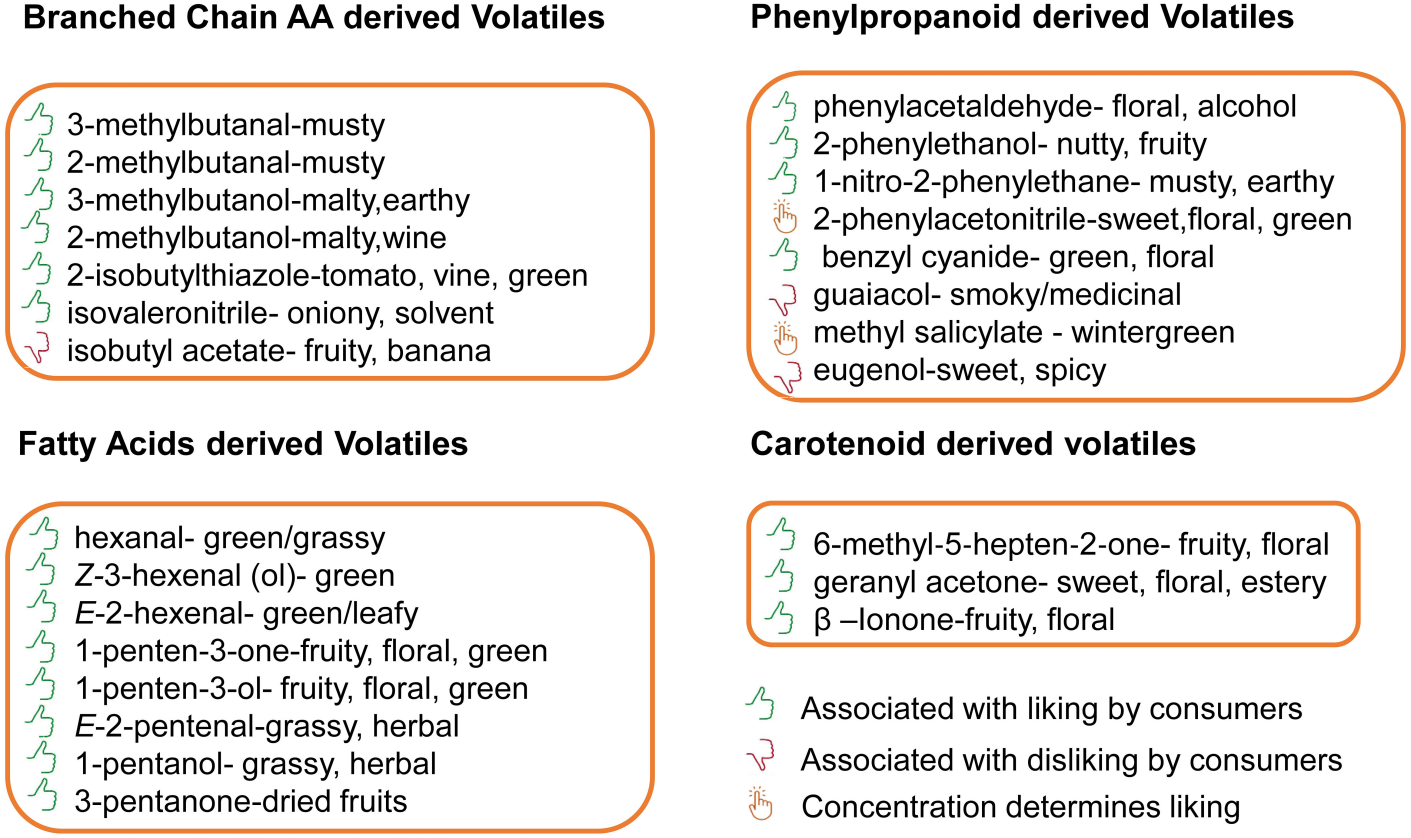
VOCs that make important contributions to flavor in tomato and their odor characteristics. The branched chain volatiles are classified as musty, malty, earthy, malty, wine, tomato vine, green, pungent flavors. The phenylalanine denved volatiles are notable for floral alcohol, nuttyfruity, musty, earthy or as smoky, medicinal and wintergreen flavors. The apocarotenoids are know to be fruity floral and sweet floral while the fatty acids demved volatiles are known for their green-grassy, tomato-green, green-leafy, fruity-flora, herbal, and dried fruit characteristics. The icons show the compounds whose odor characteristics are liked, disliked or likeness is detenmined by their concentration.

The BCAA derived volatiles are an important group of VOCs in most fruits and vegetables (Schwab et al., 2008). In tomato, the most important branched chain volatiles (BCVs) include the alcohols (3-methyl-1-butanol and 2-methyl-1-butanol), aldehydes (3-methylbutanal and 2-methylbutanal), esters (isobutyl acetate), nitriles (isovaleronitrile), and thiazoles (2-isobutylthiazole). These VOCs are important to breeders not only because they contribute to favorable flavor notes (**Figure 1**) that appear to intensify the aroma of a fresh tomato (Kazeniac and Hall, 1970; Wang et al., 2016), but also because some of them may result in off-flavors (Bizzio et al., 2022).

The pathways involved in the biosynthesis of BCAA-derived volatiles have been well characterized in microorganisms such as yeast and bacteria. In many fruits including tomato, researchers have demonstrated that some steps in the biochemical pathway to BCAA-derived volatiles are in common with the yeast and bacterial pathways (Maloney et al., 2010). Studies have shown that the breakdown of BCAAs is involved in respiration during tomato fruit ripening, but not in the production of volatile compounds in tomatoes (Kochevenko and Fernie, 2011; Kochevenko et al., 2012). In other fruits, studies have shown that BCAA transaminases are the major enzymes involved in the catabolism of BCAAs to various aroma compounds in melon (Gonda et al., 2010). However, in apples, a biosynthetic pathway called the “citramalate pathway” uses pyruvate and acetyl-CoA to form citramalic acid and, through a series of repeated reactions, leads to the synthesis of both alpha-keto acids and straight and BCV esters (2-methylbutanoate and methyl propanoate) (Sugimoto et al., 2021). The proposed pathway in tomato starts with a reversible conversion of BCAAs (leucine, isoleucine, and valine) into α-ketoacids through a deamination or transamination reaction catalyzed by branched-chain amino acid aminotransferases (BCATs) (Maloney et al., 2010; Qi et al., 2011; Ameye et al., 2018). α-ketoacids are then metabolized by three different pathways to form volatile aldehydes by α-ketoacid decarboxylase; α-hydroxyacids are formed by the action of an α-hydroxyacid dehydrogenase; aldehydes are released through the action of α-ketoacid decarboxylase, which could be reduced to an alcohol by alcohol dehydrogenases. However, Kochevenko et al. (2012) report that α-ketoacids, not amino acids, are the precursors for BCAA volatile synthesis.

**Figure 2:**
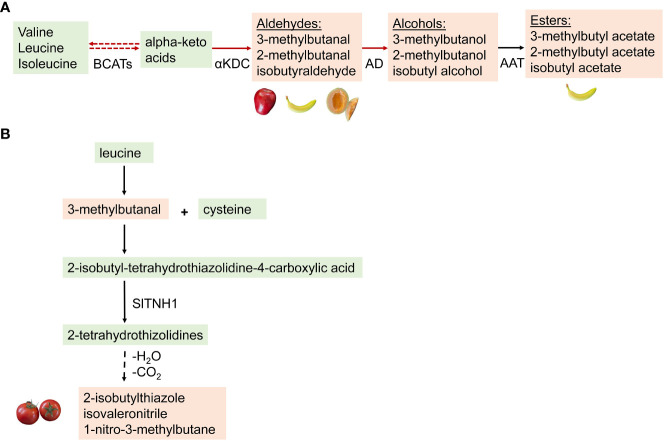
**(A)** A hypothetical biosynthetic pathway for conversion of branched chain amino acids to volatiles. Green box: Non-volatile precursors and intermediates; orange box: volatile compounds formed. Red lines indicate pathways happening in microorganisms such as yeast and bacteria, while dashed ines represent pathways unknown in tomatoes. Many enzymes are involved in the proposed pathway- BCATs, branched-chain amino acid aminotransferases; AD, Aldehyde dehydrogenases; αKDC, α-ketoacid decarboxylase; AAT, alcohol acyltransferase; CXE, carboxyesterase. **(B)** Proposed pathway for the synthesis of the BCV 2-sobutylthiazolein Tomato. The aldehyde fonmed from the breakdown of leucine, conjugates with a cysteine to form a substituted thizolidine by the action of tetrahydrothiazolidine N-hydroxylase (SITNHI) (Liscombe et al., 2022). This is followed by a series of hydroxylation and decarboxylation reactions to form 2-sobutylthiazole. Dotted lines represent reactions where the catalytic enzyme is unknown.

Alcohols can be converted to a volatile ester by the action of an alcohol acyltransferase (**Figure 2A**). Enzymatic characterization of SlAAT1 in tomato revealed that the enzyme could use a wide range of alcohols as substrates. The tomato enzyme showed the highest specific activity with 2-methyl-1-butanol. A comparison of AAT1 from tomato and the closely related species, *S. pennellii*, reveals that the *S. pennellii* enzyme (SpAAT1) is more active overall than its tomato counterpart. The most significant difference in activity was measured with 3-methyl-1-butanol and butanol. The generalized increase in activity for all the alcohols tested is particularly noticeable, given the structural diversity of the substrates. SpAAT1 also retains activity with longer acyl-CoAs, while the activity of SlAAT1 decreases quickly as the length of the chain increases (Goulet et al., 2015). Esters can be converted to alcohols by a carboxyesterase (CXE) (Kochevenko and Fernie, 2011; Goulet et al., 2012; Kochevenko et al., 2012; Rambla et al., 2017). Volatile esters have been reported in many soft fruit species during ripening, including apple, pear, banana, strawberry, kiwifruit, pineapple, and melon (Beekwilder et al., 2004). In some fruits like blueberry and tomato, volatile esters are found, which negatively contribute to flavor. A study has reported relatively lower levels of volatile esters in tomato controlled by high expression of an esterase gene (*SlCXE1*) (Goulet et al., 2012).

Among the BCAA-derived volatiles in tomato are several nitrogenous compounds, including 2-isobutylthiazole, isovaleronitrile, and 1-nitro-3-methylbutane, whose biosynthesis was recently shown to be biosynthesized from a BCAA precursor. Liscombe et al. (2022) proposed that the biosynthesis of these aroma compounds starts with the conversion of leucine to 3-methylbutanal. The aldehyde 3-methylbutanal undergoes a conjugation with cysteine to generate a substituted thiazolidine, 2-isobutyl-tetrahydrothiazolidine-4-carboxylic acid followed by sequential hydroxylations to generate the nitrogenous volatiles (**Figure 2B**).

### 2.2 Phenylpropanoid-derived volatiles

The major phenylalanine‐derived volatiles found in tomato fruit include phenylacetaldehyde, 2-phenylethanol, 1-nitro-2-phenylethane, and 2-phenylacetonitrile. These volatiles make an important contribution to desirable aroma and flavor in tomatoes, and consumer panels have shown that these four volatiles are important for good flavor in tomatoes (Tieman et al., 2012; Liscombe et al., 2022). 2-Phenylethanol and phenylacetaldehyde add a pleasant flowery or rose aroma to many fruits and food products including wines, liquors, and other alcoholic beverages. In tomato, these fruity/floral notes are considered desirable by consumers; however, elevated levels of phenylethanol and phenylacetaldehyde have also been associated with undesirable flavor in tomato fruit (Tadmor et al., 2002; Tieman D. et al., 2006). High levels of these compounds give off a nauseating, unpleasant odor, while low levels (concentrations >0.005 ppm) have a sweet floral note (Petro-Turza, 1986; Liscombe et al., 2022). Therefore, the concentrations of these two volatile compounds and the balance with other volatile compounds are critical for good flavor in tomatoes.

Phenylalanine-derived volatiles are biosynthesized from phenylalanine. Aromatic amino acid decarboxylases (AADCs) act as the first step in their biosynthesis, converting phenylalanine to phenethylamine (Tieman D. et al., 2006). The *AADC* genes were identified using *Solanum pennellii* introgression lines (Eshed and Zamir). Tadmor et al. (2002) compared the phenylacetaldehyde and 2-phenylethanol levels in three lines with an introgressed region of chromosome 8 (IL8-2) from *S. pennellii* in a *Solanum lycopersicum* background, tomato variety M82. They found that IL8-2 contained up to 60-fold higher levels of 2-phenylethanol, compared to M82. Tieman D. et al. (2006) elucidated the first step in the pathway to the biosynthesis of these volatiles *in vitro* by *E. coli* expression of the protein and enzymatic analysis and *in vivo* by over- and underexpression in transgenic tomatoes.

**Figure 3:**
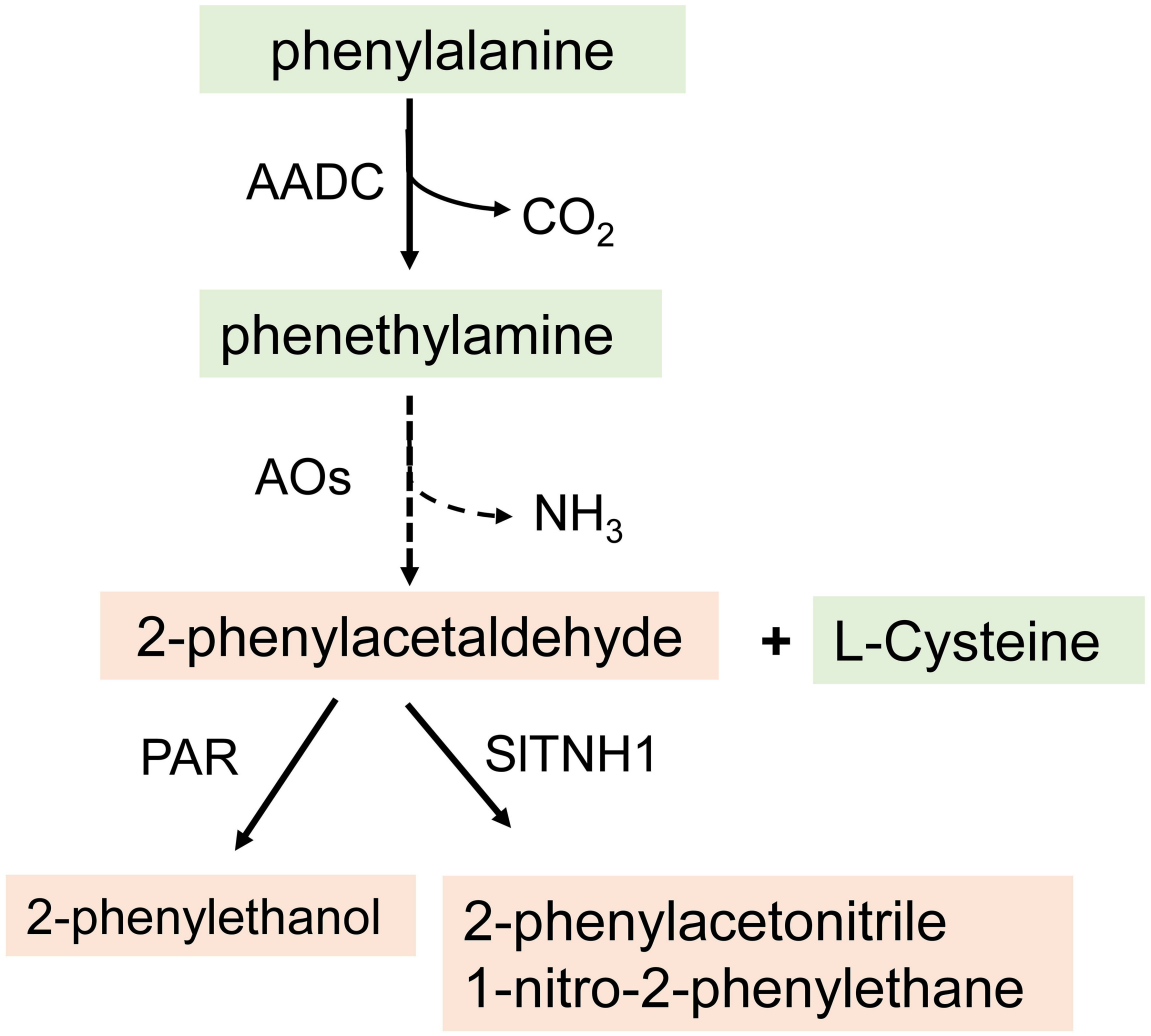
The pathway for the biosynthesis of phenylalanie-derived volatiles in tomato. AADC, aromatic acid decarboxylases; PAR, phenylacetaldehyde reductasel; AO, amine oxidase; S1TNH1, tetrahydrothiazolidine-4-carboxylic acid N-hydroxylase.

Their findings showed that phenylalanine is decarboxylated to phenethylamine by a family of aromatic amino acid decarboxylases (LeAADC1A, LeAADC1B, and LeAADC2). Phenethylamine is then converted to phenylacetaldehyde by removal of the amine group by an unknown enzyme (Wang et al., 2016) or to 2-phenylacetonitrile or 1-nitro-2-phenylethane by the action of a flavin-dependent monooxygenase enzyme (tetrahydrothiazolidine-4-carboxylic acid N-hydroxylase) using cysteine and the volatile aldehyde phenylacetaldehyde as a substrate (Liscombe et al., 2022). Furthermore, phenylacetaldehyde is converted to 2-phenylethanol by tomato phenylacetaldehyde reductases (PAR), encoded by *LePAR1* and *LePAR2* as shown by overexpression of the tomato *LePARs* in petunia flowers (**Figure 3**) (Tieman D. M. et al., 2006; Wang et al., 2016). In petunia and rose, an alternative pathway for the formation of phenylacetaldehyde has been identified. Phenylacetaldehyde synthase (PAAS) is a bifunctional enzyme that catalyzes a combined phenylalanine decarboxylation coupled to an amine oxidation reaction to generate phenylacetaldehyde (Kaminaga et al., 2006; Farhi et al., 2010).

**Figure 4:**
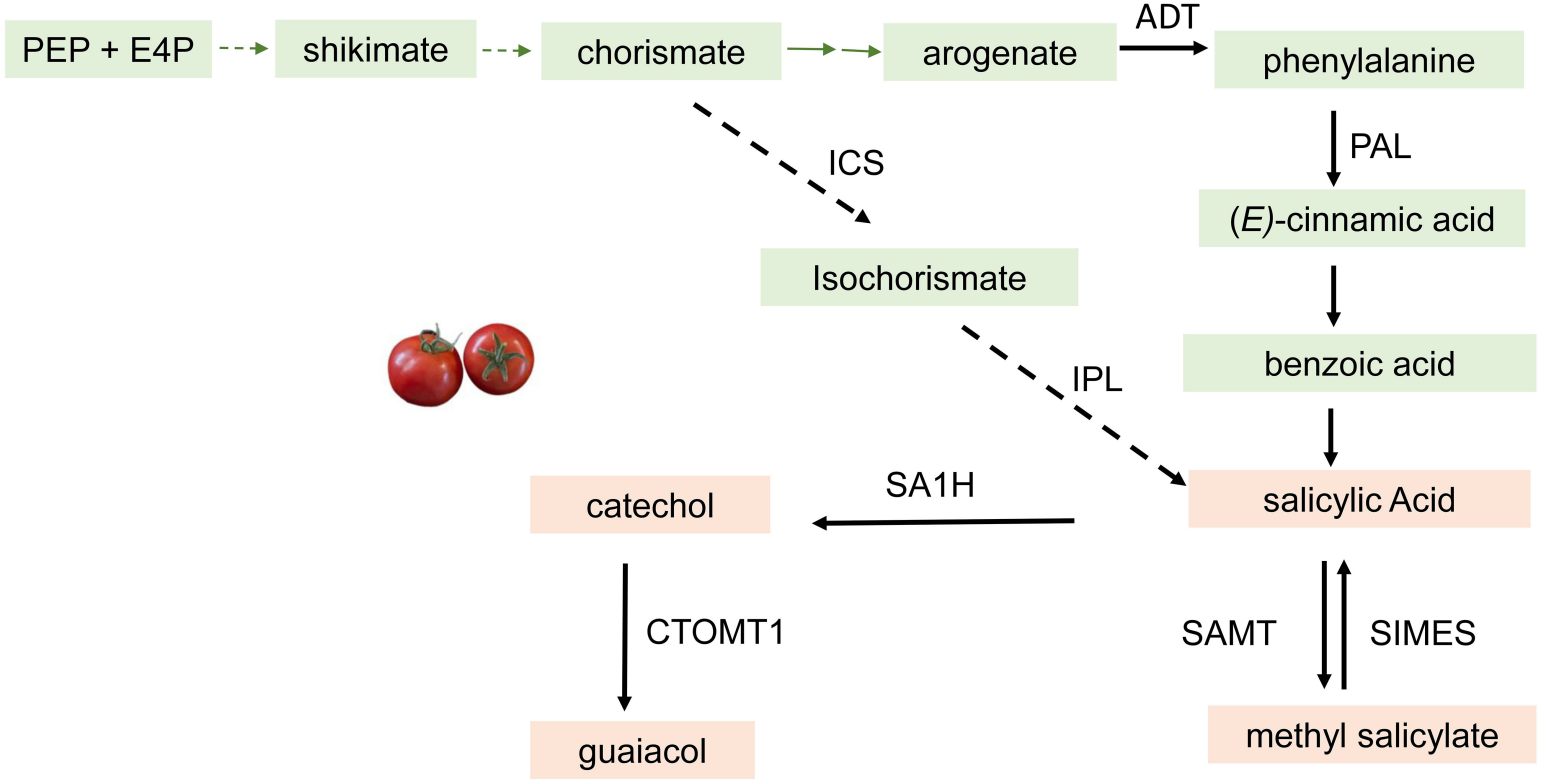
Summarized biosynthetic pathway for the Phenylpropancid derived volatiles in Tomato. The characterized enzymes that catalyze each step are shown. ICS, isochorismate synthase; IPL, isochonismate pyruvate lyase; ADT, aogenate dehydraase; CTOMT1, catechol -0- methyltranferase; SAMT, salicylic acid methyl transferase; PAL, phenylalanine ammonia lyase; SIMES, methyl esterase; SA1H, slcylic acid hydroxylase. The dashed arrows incicate steps In the pathway that have not been fully elucidated.

Phenylpropanoid-derived volatiles (PhP-V) are another important class of VOCs sharing the same phenylalanine precursor. The PhP-Vs include guaiacol, methyl salicylate (MeSA), and eugenol, which are generally described as medicinal, smoky, or pungent aromas. These compounds contribute negatively to consumer liking of the tomato fruit (Zanor et al., 2009; Tieman et al., 2012; Tieman et al., 2017). The first step of this biosynthetic pathway is a reaction between phosphoenolpyruvate (PEP) and erythrose 4-phosphate (E4P) through a series of steps to form chorismate followed by conversion to arogenate. Arogenate is then converted to phenyalanine by the action of arogenate dehydratase (Dal Cin et al., 2011; Maeda et al., 2011). Phenylalanine is converted to (*E*)-cinnamic acid by phenylalanine ammonia-lyase followed by conversion to salicylic acid with benzoic acid as an intermediate. In an alternate pathway, chorismate is converted to isochorismate by isochorismate synthase followed by conversion to salicylic acid by isochorismate pyruvate lyase (Chen et al., 2009). In tomato, salicylic acid is converted to catechol by an FAD/NADH-dependent SA 1-hydrolase, SlSA1H (Zhou et al., 2021). Guaiacol is then biosynthesized by the methylation of catechol by the enzyme catechol‐O‐methyltransferase CTOMT1 (Mageroy et al., 2012). This enzyme has been identified and characterized for its ability to produce guaiacol from catechol *in vitro*. However, it may not be the rate-limiting step to guaiacol biosynthesis in tomatoes. Transgenic plants that overexpress the *CTOMT1* gene resulted in slightly higher guaiacol production, while knockdown of *CTOMT1* resulted in lower guaiacol emission (Mageroy et al., 2012; Wang et al., 2016). Ripe tomato pericarp discs from the *CTOMT1*-overexpressing plants could convert exogenously applied catechol to guaiacol. This result confirmed that the availability of catechol probably limits guaiacol synthesis in tomato fruit tissue (**Figure 4**). Methylation of SA is catalyzed by salicylic acid methyltransferases (SAMTs) in tomato fruit (Tieman et al., 2010). Methyl esterases (*SlMES1–4*) are shown to reduce methyl salicylate levels by converting to salicylic acid in tomato (Frick et al., 2022) (**Figure 4**).

Since MeSA is involved in a dual role as a defense chemical compound and a volatile compound that negatively influences consumer liking, breeders are challenged on how to retain the disease resistance trait while reducing the flavor volatile traits. SA is a phytohormone that is involved in the plant defense system specifically as a signal for induced resistance in plants, which is also known as systemic acquired resistance (SAR) (Métraux, 2002). Overall, SA biosynthesis-related genes involve multiple levels of control in their transcripts. Some transcription factors, such as ETHYLENE INSENSITIVE3 (EIN3) and ETHYLENE INSENSITIVE3 LIKE1 (EIL1), negatively regulate SA synthesis (Li et al., 2019). Plants tightly control the relative quantities of SA and MeSA through various biochemical alterations, including methylation of SA to form MeSA and glycosylation of MeSA to prevent emission.

### 2.3 Carotenoid-derived volatiles (apocarotenoids)

The apocarotenoid volatiles are generally described as having fruity/floral aroma notes and have positive effects on the overall human appreciation of tomato (Tieman et al., 2012). The most common apocarotenoids in tomato fruit are 6‐methyl‐5‐hepten‐2‐one, geranylacetone, and β-ionone. Despite the important contribution they make to flavor, their levels of some of these compounds are lower in modern tomato varieties than in heirloom tomatoes. Studies showed that this is because some versions of alleles responsible for their synthesis were selected against during the tomato improvement process, thereby leading to reduced flavor in modern varieties (Tieman et al., 2017; Klee and Tieman, 2018; Gao et al., 2019; Zhao et al., 2019). For a long time, breeders focused on selecting deep-red uniform fruits. In the early 20th century, the *uniform* mutation, which greatly reduces the unattractive green-shoulder phenotype resulting in a uniformly red fruit, was identified, and today, the *mutant* uniform allele is present in virtually all modern commercial cultivars (Powell et al., 2012). The uniform mutation is in a Golden 2-like Transcription Factor and results in lower sugars and carotenoids in ripe tomato fruit. Similarly, the deep red color trait, associated with high levels of lycopene, a precursor of 6‐methyl‐5‐hepten‐2‐one, was selected, resulting in higher 6-methyl-5-hepten-2-one levels in modern tomatoes. It was only recently shown that the alleles selected for in modern tomatoes resulted in higher 6‐methyl‐5‐hepten‐2‐one and lower geranylacetone levels in tomato fruit (Tieman et al., 2017). Knowledge of the chemical, sensory, and biosynthetic pathways of these compounds have created a way forward to restore lost alleles in commercial tomatoes.

**Figure 5:**
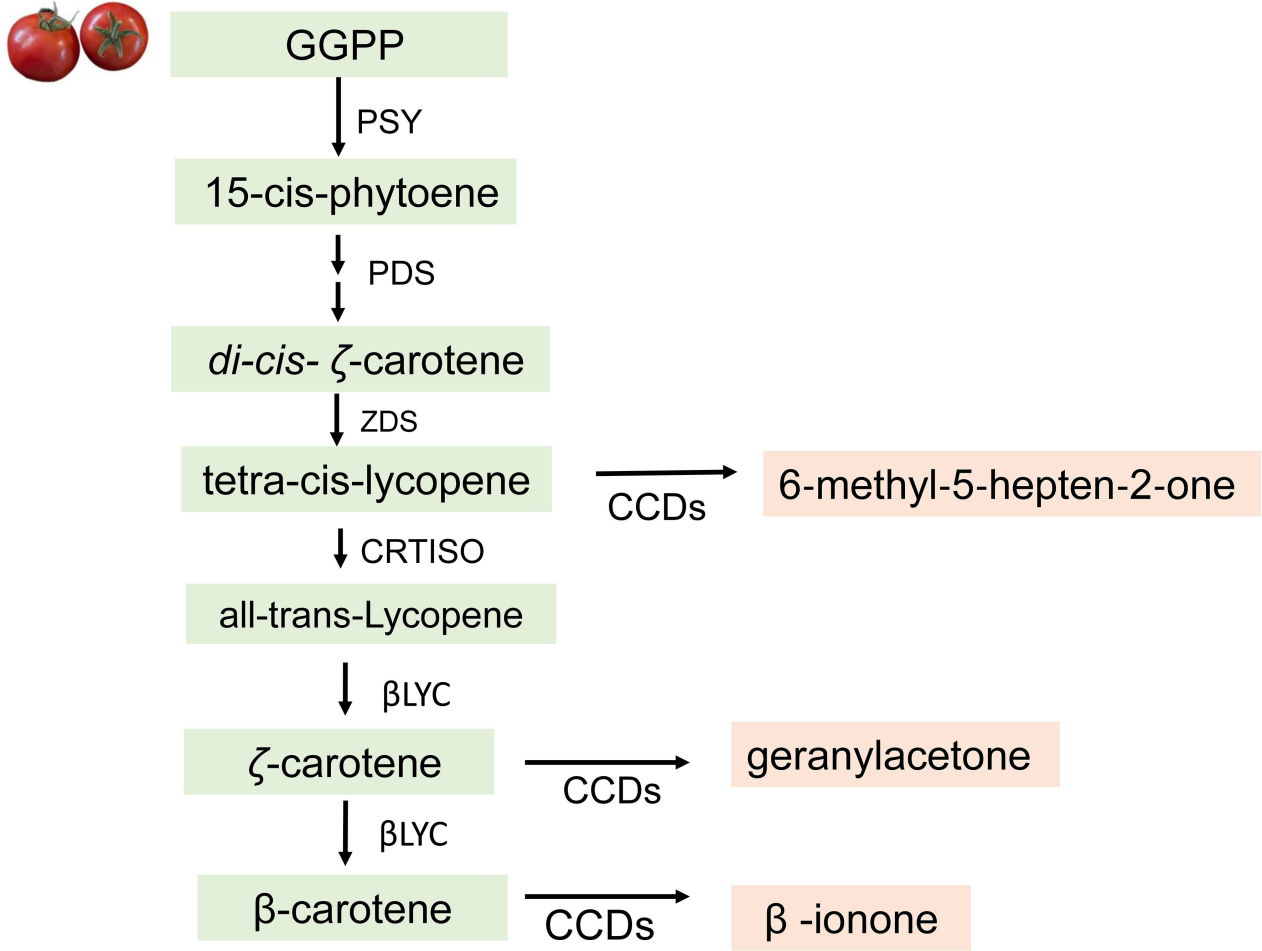
Biosynthesis of apocarotenoid volatiles in tomatoes. CCDs, carotenoid cleavage dioxygenases; GGPP, geranylgeranyl diphosphate; PSY, phytoene synthase; PDS, phytoene desaturase; ZDS, zeta-carotene desaturase; CRTISO, carotenoid isomerase; βLYC, lycopene beta-cyclase.

The biosynthetic pathway of apocarotenoids starts with an enzymatic oxidative cleavage of C40-carotenoids to lycopene, *ζ*-carotene, and β-carotene, the direct precursors of apocarotenoids (Buttery et al., 1989). In tomato, the carotenoid cleavage dioxygenase enzymes—CCDs—are involved in the synthesis of apocarotenoids during fruit ripening (Simkin et al., 2004). These CCD enzymes cleave lycopene to generate 6‐methyl‐5‐hepten‐2‐one, *ζ*-carotene to form geranylacetone, and β-carotene to generate β-ionone (Vogel et al., 2008) (**Figure 5**). However, since lycopene content is responsible for the red color of the fruit, the amount of 6‐methyl‐5‐hepten‐2‐one generated depends on its accumulation or availability. In the carotenoid biosynthetic pathway, the production of lycopene varies and can be further converted to β-carotene in tomato mutants with increased lycopene β-cyclase activity resulting in increased β-carotene and an orange-colored fruit (Ronen et al., 2000). Vogel et al. (2008) showed that mutants with altered carotenoid levels affect production of apocarotenoid volatiles and sensory properties of mutant fruit.

Numerous studies have gone into understanding the role of the CCD enzymes in the biosynthesis of apocarotenoid volatiles. (Vogel et al., 2008) described them as broad substrate specific enzymes that recognize both the linear and cyclic carotenoids. LeCCD1A and LeCCD1B cleave linear carotenoids at either the 5,6, the 7,8, or the 9,10 positions, and cyclic carotenoids at the 9,10 position to generate various apocarotenoids. LeCCD1A generates 6-methyl-5-hepten-2-one through the cleavage of lycopene at the 5,6 or 5′,6′ bond positions. β-ionone is synthesized from β-carotene by free radical-mediated cleavage of the 9-10 bond, while geranylacetone, is generated by oxidative cleavage of phytoene at the 9,10 (9′,10′) positions (Vogel et al., 2010).

Simkin et al. (2004) observed that the downregulation of the *LeCCD1A* and *LeCCD1B* genes using an antisense construct in tomato resulted in a significant reduction in the rates of emission of geranylacetone and β-ionone in cut tomato fruits, but did not affect the overall carotenoid content (Simkin et al., 2004; Cheng et al., 2021; Simkin, 2021). This suggests that additional enzymes may be involved in carotenoid cleavage. Among them are CCD4, CCD7, and CCD8 identified for their role in cleaving carotenoids at the appropriate bonds to generate apocarotenoids (Simkin et al., 2004; Cheng et al., 2021; Simkin, 2021). Although CCD1A and CCD1B are not localized to the chloroplast and are shown to be the only enzyme involved in production of flavor apocarotenoids, CCD4, CCD7, and CCD8 play other functions in plants. For example, CCD4 is localized to the chloroplast and plastoglobules where carotenoids are stored and may be involved in carotenoid turnover (Simkin et al., 2004; Cheng et al., 2021; Simkin, 2021). In Arabidopsis, CCD7 is previously reported to be involved in conversion of β-carotene into β-ionone and 10’-apo-β-carotenal by a 9’-10’ symmetrical cleavage activity (Schwartz et al., 2004). The 10’-apo-β-carotenal was further cleaved into 13-apo-β-carotenone and a C9 dialdehyde with the combined expression of AtCCD7 and AtCCD8 in *E. coli*. It was concluded that sequential cleavage of CCD7 followed by CCD8 result in the formation of 13-apo-β-carotenone (Simkin et al., 2004; Cheng et al., 2021; Simkin, 2021). Some of the activities of these enzymes have been demonstrated in other horticultural crops; however, the regulatory mechanism involved in the synthesis of apocarotenoids in tomatoes is still an area of active research.

### 2.4 Fatty acid-derived volatiles

Distinguishable for their green or grassy aromas, the fatty acid-derived volatiles make important contributions to flavor and consumer liking of tomatoes (Buttery et al., 1989; Klee, 2010). Several short- and moderate-chain, fatty acid-derived volatiles (C4–C12) are present in most fruits, but the fatty acid-derived volatiles most associated with desirable flavor in tomato include the C5 [1-penten-3-one, (*E*)-2-pentenal, 3-pentanone, 1-pentanol, and 1-penten-3-ol] and C6 [hexanal, (*Z*)-3-hexenal, (*Z*)*-*3-hexenol, and (*E*)*-*2-hexenal] short-chain volatiles. An analysis of the volatile contents and compositions of tomato fruits showed that fatty acid-derived volatiles accounted for more than half of the total volatiles, with C6 volatiles being the predominant volatiles in ripe fruit (Buttery et al., 1989; Chen et al., 2004; Klee, 2010; Tieman et al., 2012).

**Figure 6:**
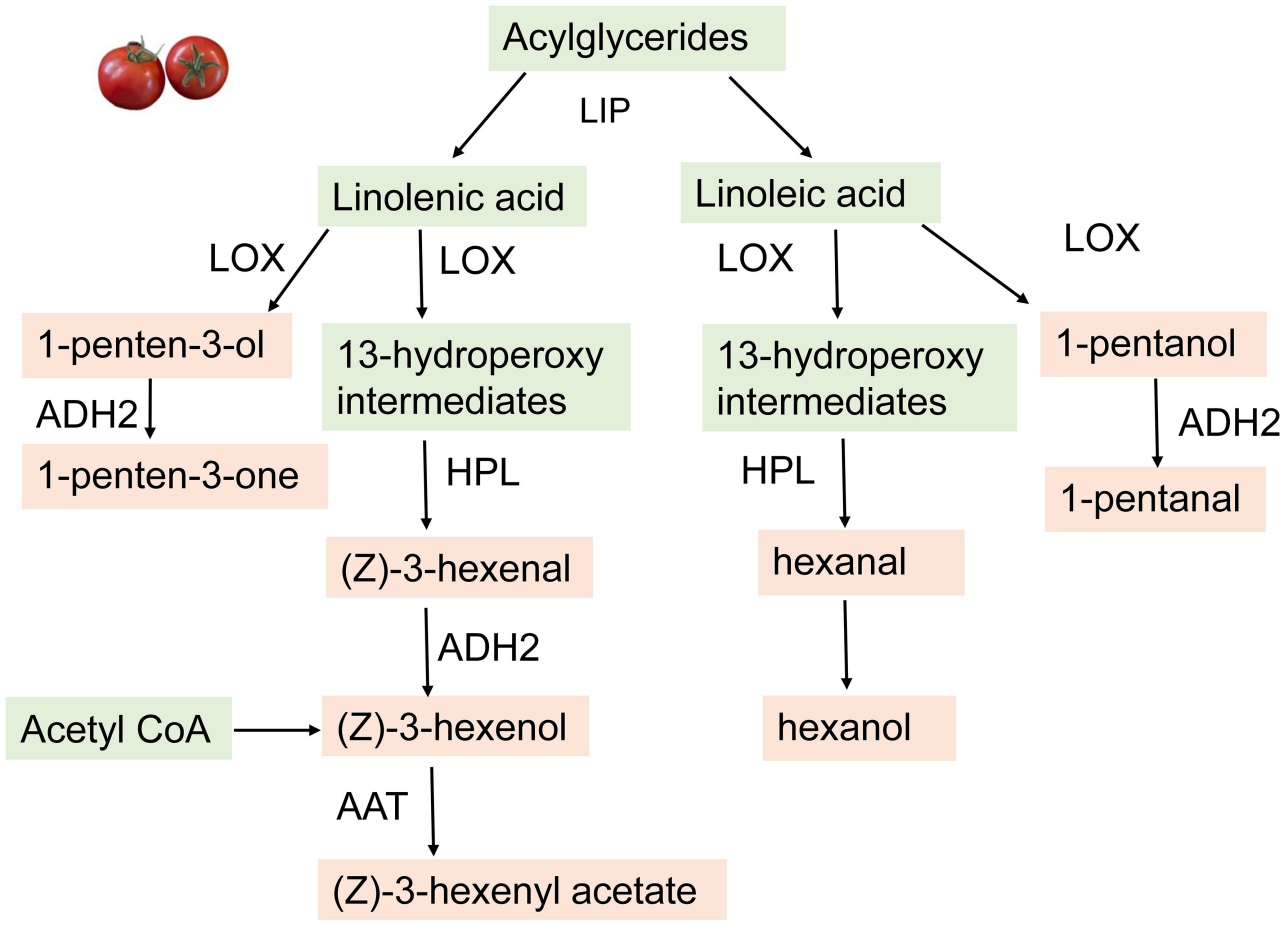
Biosynthetic mechanism for the fatty acid derived volatiles in Tomatoes. The enzymes involved include LOX, lipoxygenase; HPL, hychoperoride fyase; ADH2, alcohol dehydrogenase2 (ADH2) and AAT, alcohol acyltransferase; LIP, lipase.

The biosynthesis of these volatiles has been well studied in tomatoes. During ripening, cell disruption causes the catabolism of acylglycerides by lipases leading to the formation of fatty acids. Through a combination of a metabolite-based genome-wide association study, genetic mapping, and functional analysis, Li X. et al. (2020) identified *Sl-LIP8* as a gene involved in the accumulation of short-chain FA-VOCs. During the first step of FA-VOCs synthesis, Sl*-LIP8* cleaves acylglycerides to release linolenic and linoleic acid, the two major precursors of C6 and C5 volatiles in tomato (Li X. et al., 2020). Further oxidation of the linoleic and linolenic acids at the C13 position produces 13-hydroperoxy intermediates through the action of a 13-lipoxygenase (13-LOX). Out of the six LOX-encoding genes (*TomloxA–F*) *TomloxC* encodes the LOX enzyme that is essential for the formation of these intermediates in fruit (Chen et al., 2004). These intermediates then enter two different branches of the LOX pathway to produce the C6 volatile compounds (Chen et al., 2004) (**Figure 6**).

The hydroperoxide lyases (HPL) cleaves the 13-hydroperoxy (13HPOs) derivatives to produce two short-chain C6 aldehydes including hexanal, (*E*)-2-hexenal, and (*Z*)*-*3-hexenal. The final step in the process of C6 FA VOCs biosynthesis is the conversion of the C6 aldehydes to alcohols. The conversion of aldehydes to alcohols is catalyzed by the alcohol dehydrogenase2 (ADH2) enzyme (Klee, 2010). For example, the overexpression of an *ADH2* gene led to increased levels of hexanol and (*Z*)*-*3-hexenol in tomato, a phenotype that consumers described as having a more intense “ripe fruit” flavor (Schwab et al., 2008; Ameye et al., 2018). However, studies conducted in Arabidopsis have shown that NADPH-dependent aldehyde- or aldo-keto reductases are also involved in this process (Ameye et al., 2018). Furthermore, alcohol acyltransferase (AAT) modifies the alcohols to their corresponding esters. AAT catalyzes the formation of (*Z*)*-*3-hexenyl acetate or hexyl acetate from (*Z*)*-*3-hexenol and hexanol, respectively (Schwab et al., 2008; (Goulet et al., 2015).

The mechanism involved in the synthesis of the C5 FA VOCs in tomato has been an active area of research. Shen et al. (2014) reported that the tomato 13-lipoxygenase (*TomLoxC*) is one of the major enzymes involved, and reduction of *TomLoxC* expression results in lower levels of C6 and C5 volatiles, including (*Z*)*-*3-hexenal, hexanal, hexanol, (*Z*)*-*3-hexen-1-ol, 1-penten-3-ol, 1-penten-3-one, pentanal, (*Z*)*-*2-penten-1-ol, and 1-pentanol. However, downregulation of a tomato hydroperoxide lyase (HPL) decreases C6 volatiles and increases C5 fruit volatiles. In other crops like soybean, the pathway for the synthesis of these C5 volatiles was proposed to be catalyzed by two separate LOX reactions leading to the generation of a hydroperoxide and then an alkoxyl radical. The alkoxyl radical would undergo non-enzymatic cleavage to generate C5 alcohols. The alcohols can then be reduced to their corresponding aldehydes by alcohol dehydrogenases (Salch et al., 1995). Tomé-Rodríguez et al. (2021) also reported a similar pathway in olives, even though the mechanism is not fully elucidated.

C9 volatiles such as a (*E,Z*)-2,6-nonadienal are important flavor volatiles in cucumber fruit, but are not significant contributors to flavor in cultivated tomato. Both 9-lipoxygenases that catalyze the conversion of fatty acids to 9-hydroperoxides and 9/13-lipooxygenases that can cleave fatty acids to form 9- and 13-hydroperoxides have been identified in plants. A tomato introgression line with a portion of chromosome 1 from *Solanum pennelli* in a *S. lycopersicum* background had high levels of C9 volatiles. A 9/13-hydroperoxide lyase found on the introgression was shown to be responsible for the formation of C9 volatiles, and the *S. lycopersicum* homolog of this protein lacked an amino acid residue critical for HPL activity (Matsiu et al., 2007).

Garbowicz et al. (2018) conducted a metabolic quantitative trait loci (mQTL) and lipidomic profile analysis in 76 *S. pennelli* tomato introgression lines, and identified a lipase (LIP1) that catalyzes the synthesis of (*Z*)-4-decenal, (*E*, *E*)-2,4-decadienal, and (*E*)-2-octenal, long-chain fatty acid-derived volatiles that make important contribution to liking and flavor intensity. The authors noted that this lipase degrades triacylglycerols to produce multiple fatty acid-derived flavor volatiles, and that the levels of some of these may be increased by selecting lines with higher expression of the enzyme in tomato.

### 2.5 Glycosylation

**Figure 7:**
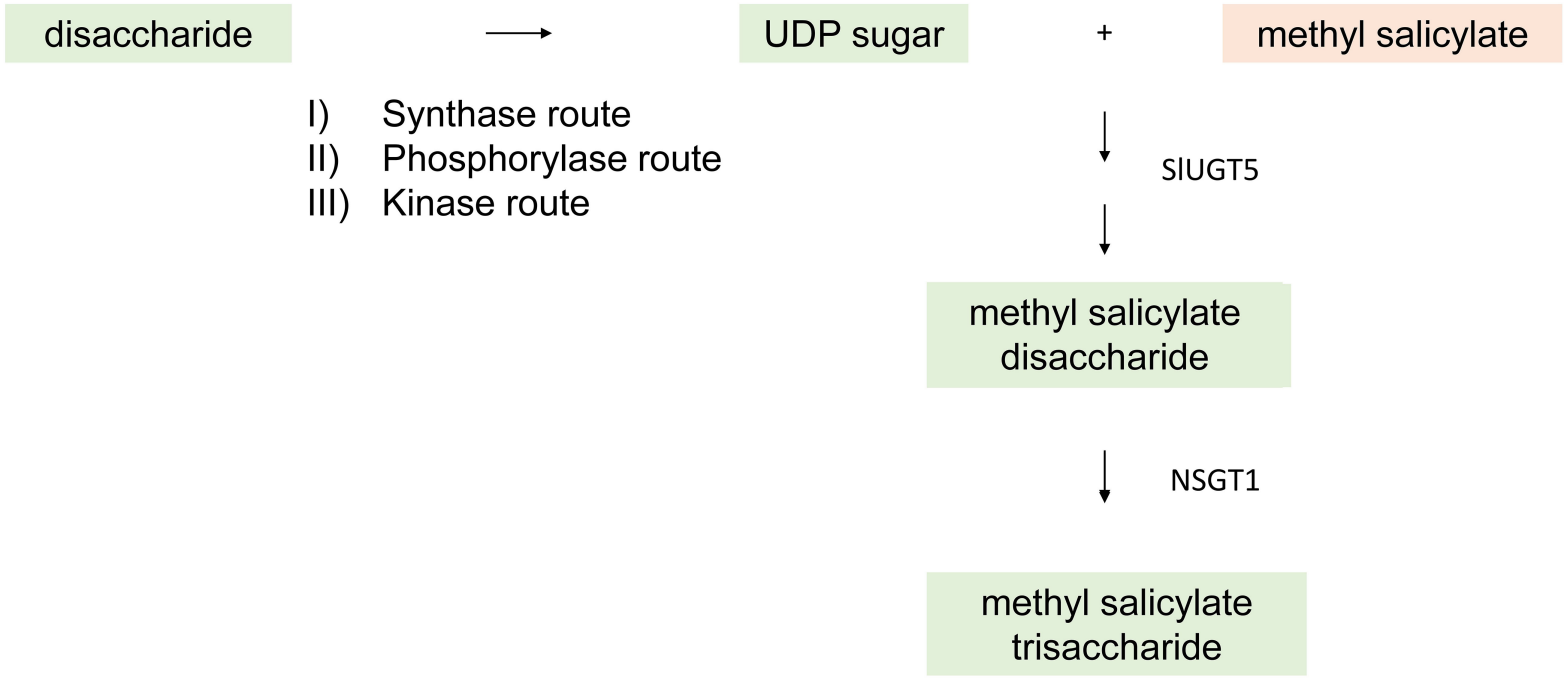
General overview of a glycosylation reaction catalyzed by UDP-glycosyltransferases (UGTs) by transferring sugar molecules to the VOC by three possible routes - synthase, phosphorylase, and kinase route. An example of the glycosylated VOC methyl salicylate is demonstrated catalyzed by SIUGTS and NSGT1.

Many secondary metabolites, including flavor VOCs, are glycosylated in plants. Glycosylated VOCs do not contribute to aroma. The glycosylated VOCs are glycosides composed of a glucopyranosyl unit attached through a β-glycosidic linkage to an aglycone. This conjugated volatile form is odorless, but the free volatile can be released under acidic conditions or enzymatically by glycosylases. The glycoside forms are stored in vacuoles. Glycosylation reactions are usually catalyzed by UDP-glycosyltransferases (UGTs) by transferring sugar molecules to the VOC (**Figure 7**) (Bönisch et al., 2014). Nucleotide sugars are typically used as activated donors in glycosylation reactions and uridine diphosphate (UDP) sugars are the most common (De Bruyn et al., 2015). UDP sugars can be biosynthesized by three different pathways. The synthase pathway produces a UDP sugar in a reversible reaction directly from a disaccharide, usually sucrose in plants. The phosphorylase pathway produces an activated sugar 1-phosphate by cleaving disaccharides with inorganic phosphate as a cofactor. The phosphorylated sugar and UTP are then acted on by an uridylyl transferase to make corresponding UDP sugar. The most prominent phosphate sugar is α-glucose 1-phosphate. The third route is a kinase route involving the action of both kinase and uridylyl transferase to generate UDP sugar (Kleczkowski et al., 2010; De Bruyn et al., 2015).

Glycosylation can change phenylpropanoid solubility, stability, toxicity, compartmentalization, and biological activity. Hundreds of UGT genes can be found in a plant genome, but the substrates of very few UGTs have been identified. UGT genes responsible for the formation of glycosylated volatiles have been characterized in tomato (Louveau et al., 2011; Tikunov et al., 2013), grape (Bönisch et al., 2014), kiwifruit (Yauk et al., 2014), peach (Wu et al., 2019), and strawberry (Song et al., 2016). These UGTs are found to be involved in the metabolism of various substrates including the monoterpenols geranial, *R,S*-citronellol, nerol, and *R,S*-linalool in grapes (Bönisch et al., 2014); geraniol, linalool, octan-3-ol, 2-phenylethanol, and hexanol in kiwi (Yauk et al., 2014); and furanone substrates in strawberry. A single UGT can glycosylate multiple structurally diverse substrates, and multiple UGTs can also glycosylate the same substrate. Therefore, substrate availability in cells can be a determining factor of the product spectrum (Tiwari et al., 2016).

The phenylpropanoid pathway in plants involves the synthesis of many secondary metabolites derived from phenylalanine and tyrosine. Phenylpropanoid volatiles (PPVs) such as guaiacol and methyl salicylate significantly contribute to tomato fruit aroma. A tomato UGT, SlUGT5, showed broad substrate specificity, and the *SlUGT5* gene was mainly found in fruits and flowers. SlUGT5 had activity on the flavor VOCs guaiacol, eugenol, benzyl alcohol, and methyl salicylate, as well as hydroquinone and salicyl alcohol (Louveau et al., 2011). Guaiacol imparts a “smoky” or “pharmaceutical” flavor in tomato fruit, and methyl salicylate contributes a wintergreen component to flavor. A glycosyltransferase, *NON-SMOKY GLYCOSYLTRANSFERASE1 (NSGT1)*, encoded a protein responsible for producing non-cleavable tri-glycosides from cleavable di-glycosides. Phylogenetic analysis showed that NSGT1 belongs to a specific clade of branch-forming glycosyltransferases that are involved only in elongation of the glycosidic moiety of glycosides. It was reported that structural variants at the NSGT locus are responsible for variation in PPV levels (Alonge et al., 2020). Active forms of NSGT1 are expressed during tomato fruit ripening and varieties with active forms of the enzyme had lower levels of guaiacol, while haplotypes without functional NSGTs had higher guaiacol levels. It was found that NSGT1 could not add glycosides to the aglycone form of PPVs, but only added to the elongation of glycosides (Tikunov et al., 2013). A knockout mutation of NSGT1 in “smoky” tomato fruits resulted in the accumulation of guaiacol β-primerverosides and guaiacol can be released. However, in “non-smoky” tomato fruits, a functional NSGT1 converts β-primerverosides to 2-O-β-d-glucopyranosyl-(1→2)-[O-β-d-xylopyranosyl-(1→6)]-O-β-d-glucopyranoside and guaiacol cannot be released.

In peach, the monoterpene linalool is a significant contributor to aroma and flavor (Wu et al., 2019). Approximately 40% of linalool in ripe peach fruit is present in a non-volatile glycosylated form affecting overall fruit flavor. It was reported using RNA sequencing, subcellular localization, and transient expression that *PpUGT85A2* is a key regulator controlling linalyl-β-D-glucoside content in peach. Glycosylation activities during ripening were found to be different in diverse genotypes of grapes. In grapes, two glycosyltransferase genes involved in the metabolism of monoterpenols *in vivo* were identified using an activity-based metabolic profiling (ABMP) approach (Duckworth and Aldrich, 2010; Bönisch et al., 2014) of a grape berry aglycone library. ABMP allows for testing of broad substrate preferences of UGTs, which otherwise require many substrates to be tested. ABMP is based on the application of chromatographic techniques to understand the impact of a recombinant enzyme on the homologous cellular extract representing potential substrates and products. In strawberries, HDMF [4-hydroxy-2,5-dimethyl-3(2H)-furanone] is an important volatile responsible for fruit flavor. HDMF, homologs of HDMF, i.e., EHMF [tautomeric 2 (or 5)-ethyl-4-hydroxy-5 (or 2)-methyl-3(2H)-furanone, homofuraneol], and HMF (4-hydroxy-5-methyl-3-furanone, norfuraneol) were tested as substrates for several UGTs. It was found that along with HDMF, EHMF was glycosylated by the UGTs (Song et al., 2016).

### 2.6 Regulation of gene expression

Many tomato flavor volatiles increase with ripening, with low levels in green fruit and high levels in ripe fruit (Tieman D. M. et al., 2006). Several transcription factors (TFs) regulate genes involved in fruit ripening and flavor-related pathways including RIPENING INHIBITOR (RIN), COLORLESS NON-RIPENING (CNR), TOMATO AGAMOUS-LIKE1 (TAGL1), APETALA2a (AP2a), NON-RIPENING (NOR), and FRUITFULL (FUL1 and FUL2) (Wang et al., 2019). The *RIN MADS* box TF controls volatile aldehyde and alcohol production by regulation of respective biosynthetic genes in tomato (Qin et al., 2012). In peach and apple, a NAC TF regulates ester production by activating *AAT1* gene expression at the fruit ripening stage (Cao et al., 2021). Another SQUAMOSA PROMOTER BINDING PROTEIN family TF, *Cnr*, is regulated by changes in promoter methylation (Klee and Giovannoni, 2011).

DNA methylation is an epigenetic regulation that is involved in transcriptional regulation, development, stress responses, and genome integrity. In plants, DNA methylation occurs at the cytosine residues in three different sequences (CG, CHG, and CHH, where H = A, C, or T) (Cokus et al., 2008). DNA methylation is associated with inactive transcription in plants, i.e., silencing (Lang et al., 2017). In plants, cytosine DNA methylation occurs through an RNA-directed DNA methylation pathway (RdDM). The cytosines can be methylated by the DNA methyltransferase domains rearranged methyltransferase 2 (DRM2) mediated by 24-nucleotide siRNAs. DNA methylation can be maintained during replication by DNA methyltransferases or RdDM. Cytosine methylation is regulated by DNA methylation and demethylation reactions. The major reasons for loss of DNA methylation include passive DNA demethylation, which represents failure in maintaining methylation after replication, or active DNA demethylation, which includes active removal of the methyl groups by enzymes (Lang et al., 2017). Passive DNA demethylation occurs in dividing cells, when overall methylation levels after each cell division are reduced by inhibition or dysfunction of DNA methyltransferase 1 (Dnmt1) (Moore et al., 2013). An enzyme for active DNA demethylation (DEMETER-like DNA demethylases 2, SlDML2) has been characterized in tomato. SlDML2 is a homolog of the Arabidopsis ROS1 family of bifunctional 5-methylcytosine DNA glycosylase/lyases. DNA hypermethylation is prevented by the enzymes and regulatory factors involved in DNA demethylation and is regarded as a factor in anti-silencing (Lang et al., 2017). It was reported that changing tomato epigenomes could contribute significantly to fruit ripening (Zhong et al., 2013). Tomatoes treated with the methyltransferase inhibitor 5-azacytidine ripened prematurely. Furthermore, 52,095 differentially methylated regions were identified by whole genome bisulfite sequencing on tomato fruits from four development stages. DNA methylation plays a vital role in fruit ripening, particularly in *rin* binding promoter regions. The *rin* mutation is recessive and the hybrids are the foundation of modern long shelf-life tomatoes (Giovannoni, 2007). It was found that the RIN binding site is localized in demethylated regions of promoters of various ripening genes and binding happens in accordance with demethylation (Zhong et al., 2013).

DNA demethylation also contributes to ripening as a global loss of DNA methylation was observed in tomato and strawberry, suggesting that DNA hypomethylation is vital for fruit ripening. Increased expression of a DNA demethylase in tomato, SlDML2, an ortholog of Arabidopsis ROS1, is found to be responsible for the demethylation of as many as ~30,000 genomic regions, which are preferentially distributed in chromosomal arms, and is required for virtually all ripening-induced DNA demethylation. The transcriptome analysis of sldml2 mutant fruits showed that SlDML2 is necessary for the activation of ripening-related genes, including RIN, genes contributing to early stages of fruit development, and genes involved in pigment and flavor compound synthesis and cell wall hydrolysis. SIDML2-mediated DNA demethylation is also associated with the repression of many genes in tomato fruit development and ripening (Lang et al., 2017).

Chilling stress results in loss of flavor and volatiles in tomato and causes significant changes in the methylation status of the genome. These changes occur in gene promoters, which contribute to fruit ripening, quality, and flavor volatiles (Zhang et al., 2016). Early evidence that DNA methylation affects fruit ripening was shown in the case of the dominant Cnr (colorless non-ripening) mutations, affecting fruit ripening. This occurs by hypermethylation within the transcriptional promoter of an SBP-box (SQUAMOSA binding protein-like) gene. Cnr mutant fruit showed inhibition of fruit softening and an absence of carotenoid and flavor compound accumulation (Manning et al., 2006). Tomato fruit undergoes demethylation of ripening-associated transcriptional promoters at the onset of ripening, indicating that the methylation state is not static and controls ripening. Another study revealed a global gain in DNA methylation during the orange stage of fruit ripening (Huang et al., 2019).

Histone H3K27 demethylase SIJMJ6 plays a positive regulatory role in tomato fruit ripening. Histone methylation, which mainly occurs at specific lysine and arginine residues located on the N-terminal tails of the core histones, plays an essential role in chromatin configuration and gene expression regulation. Histone lysine residues can be mono-, di-, or trimethylated, which are dynamically controlled by histone lysine methyltransferases and demethylases. Histone H3K27 demethylase SIJMJ6 plays a positive regulatory role in tomato fruit ripening (Liu et al., 2010). There are two known families of histone lysine demethylase, lysine-specific demethylase 1 (LSD1), and Jumonji C-terminal (JmjC) domain-containing demethylases. SlJMJ6, a member of the plant-specific KDM5/JAR2DI sub-family of JmjC domain-containing proteins, is an H3K27 demethylase with apparent demethylation activity for trimethylation at H3K27. Notably, SlJMJ6 directly activates the expression of DML2, an essential DNA demethylase that is required for fruit ripening. Overexpression of SlJMJ6 accelerates tomato fruit ripening, which is associated with the upregulated expression of ripening-related transcriptional regulation, ethylene biosynthesis and signal transduction, cell wall degradation, carotenoid biosynthesis, and flavor volatile biosynthesis genes. SlJMJ6 is a ripening-prompting H3K27me3 demethylase that activates the expression of the ripening-related genes by modulating H3K27me3, thereby facilitating tomato fruit ripening. There is a link between histone demethylation and DNA demethylation in regulating fruit ripening (Li Z. et al., 2020). Overall, the locus‐specific changes of DNA methylation could modify fruit traits both at early developmental stages and during maturation. Plant breeding strategies should consider not only genetic variation but also epigenetic variation as a source of phenotypic variations (Tang et al., 2020).

## 3 Advances in tomato flavor research

**Figure 8:**
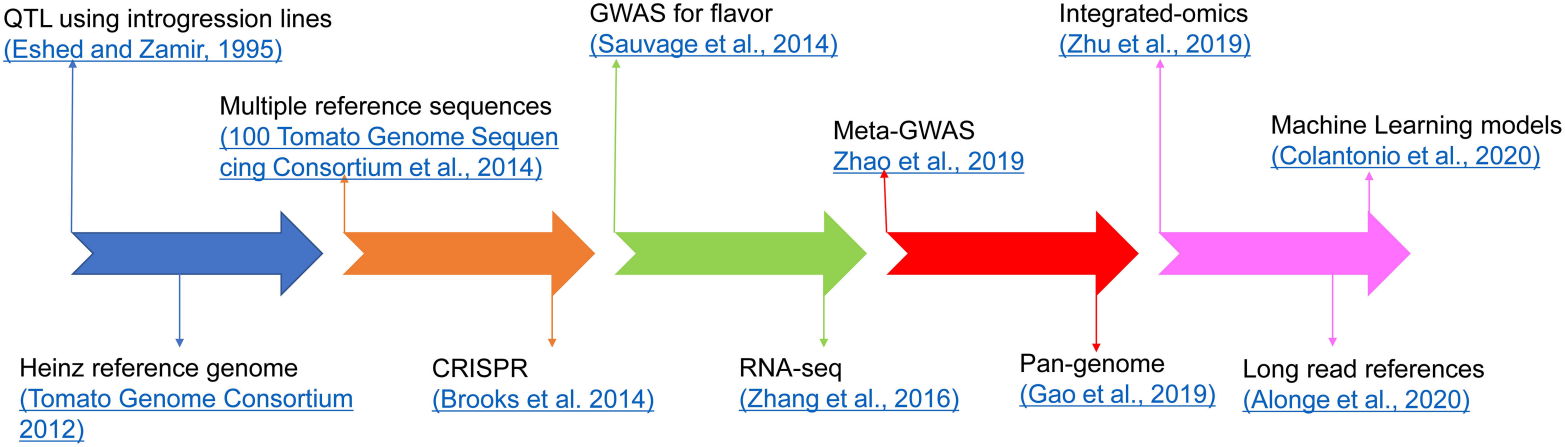
Timeline for advancements in tomato flavor research.

Several studies have shown that the flavor of tomato varieties deteriorated while selecting for other traits, such as yield, fruit size, shelf life, and disease resistance (Tieman et al., 2012; Klee and Tieman, 2013; Klee and Tieman, 2018; Zhao et al., 2019). For years, breeders focused on traits targeted at economic value and yield, and especially disease resistance, leading to loss of flavor. Flavor is very difficult to phenotype, requiring expensive instrumentation, taste panels with many people, and skilled staff to perform the testing. This has made it difficult to breed for good flavor, especially in tomato with a very complex aroma and flavor profile. Recent research has focused on restoring some of these flavor traits while ensuring that other traits of economic value are not lost. Using molecular biology, genetics, and genomics tools, some of the loci and genes involved in flavor volatile synthesis in tomatoes have been identified. We have shown a timeline here for the progress in tomato flavor research using omics technologies (**Figure 8**; **Tables 1**, **2**).

**Table 1:** QTL studies utilized in tomato flavor research.

Biochemicals	Germplasm	Loci	References
Soluble solids and fruit mass	50 *S. pennelli* ILs	23 QTLs for soluble solids	Eshed and Zamir, 1995
Flavor volatiles and citric acid	74 *S. pennelli* ILs	23 QTLs for volatiles and 4 QTLs for citric acid	Tieman D. M. et al., 2006
Flavor volatiles	89 *S. habrochaites* ILs	Identified 30 QTLs for volatiles	Mathieu et al., 2009
Flavor volatiles, sugars, acids	144 RILs from cross between a cherry tomato and a large fruited variety	Identified 81 significant associations for 26 traits	Saliba-Colombani et al., 2001
Flavor volatiles, sugars, acids, sensory attributes	144 RILs from cross between a cherry tomato and a large fruited variety	Revealed 130 QTLs for 38 traits based on IM and CIM methods.	Causse et al., 2002
Flavor volatiles	169 *S. pimpinellifolium* ILs	Identified 102 QTLs for 39 volatiles	Rambla et al., 2017
Soluble solids and pH	93 *S. pimpinellifolium* IBLs	Identified 4 QTLs for soluble solids and 2 for pH	Celik et al., 2017
Sugars and organic acids	94 *S. pimpinellifolium* IBLs	Identified 14 QTLs for sugars and 71 for acids	Çolak et al., 2020

QTL, IL (quantitative trait loci, introgression lines); QTL, RIL (quantitative trait loci, recombinant inbred lines), and QTL, IBL (quantitative trait loci, inbred backcross line).

**Table 2:** GWAS (genome-wide association studies) in tomato flavor research.

	Biochemicals	Germplasm	SNPs/markers	Number of loci identified	Methods	Reference	Superscript for figure 9
GWAS	Carotenoids, phenolics, acids, and soluble solids	96 landraces and varieties	7,720 SolCAP SNPs	20	Mixed linear model (MLM) method.	Ruggieri et al., 2014	na
GWAS	Amino acids, sugars, and acids	163 *S. lycopersicum*, *S. lycopersicum* var. *cerasiforme*, and *S. pimpinellifolium*	5,995 SNPs	44	Multilocus mixed model (MLMM) method.	Sauvage et al., 2014	1
GWAS	Flavor volatiles	174 *S. lycopersicum* var. *cerasiforme* and *S. pimpinellifolium*	182 SSR markers	125	Mixed linear model (MLM) method.	Zhang et al., 2015	na
GWAS	Sugars and acids	123 *S. lycopersicum* and 51 *S. lycopersicum* var. *cerasiforme*	128 SSR markers	58	Mixed linear model (MLM) method in TASSEL.	Zhao et al., 2016	na
GWAS	Amino acids, sugars, acids, and flavor volatiles	62 *S. pimpinellifolium*, 190 *S. lycopersicum* var. *cerasiforme* and 48 *S. lycopericum*	7,667 SolCAP and 5528 CBSG SNPs	79	Multilocus mixed model (MLMM) method.	Bauchet et al., 2017	2
GWAS	27 flavor volatiles, sugars, and acids	15 *S. pimpinellifolium*, 83 *S. lycopersicum* var. *cerasiforme* and 300 *S. lycopericum*	2,014,488 SNPs	251	Mixed-model association expedited (EMMAX).	Tieman et al., 2017	3
GWAS, meta-analysis	Flavor volatiles, sugars, acids, and amino acids	453 *S. lycopericum*, 291 *S. lycopersicum* var. *cerasiforme*, 94 *S. pimpinellifolium*	2,316,117 SNPs	305	Mixed-model association expedited (EMMAX).	Zhao et al., 2019	4
GWAS	Sugars, acids, and amino acids	171 *S. lycopersicum*, 104 *S. lycopersicum* var. *cerasiforme*, and 27 *S. pimpinellifolium*	4,180,023 SNPs	126	Mixed linear model (MLM) method in TASSEL.	Ye et al., 2019	5
GWAS	Sugars, acids, and carotenoids	18 *S. lycopersicum*, 111 *S. lycopersicum* var. *cerasiforme*, and 27 *S. pimpinellifolium*	23,797,503 SNPs	8	Mixed linear model (MLM) method.	Razifard et al., 2020	6

### 3.1 QTL for flavor

Tomato has undergone domestication and improvement, leading to a narrow genetic base. Interspecific crosses have been used to introgress desirable genomic regions from wild species into modern commercial varieties to add lost traits. QTLs (quantitative trait loci) refer to genomic regions or loci responsible for affecting a quantitative trait. Many important traits, including yield, disease resistance, and flavor, are quantitative. A quantitative trait is controlled by several genes, each with a small additive effect and influenced by the environment. Many studies reported QTLs for tomato flavor based on introgression lines and recombinant inbred lines (RILs) based on interspecific and intraspecific crosses (**Table 1**). Traditionally, the QTL studies utilized whole genome segregating populations, but the epistatic interactions in those populations make it difficult to characterize individual loci. This led to the concept of introgression lines (IL), a set of nearly isogenic lines made from multiple backcrosses after the initial interspecific cross. Each line carries a single genetically defined chromosome segment from a divergent genome in a common background (Lippman et al., 2007). The QTL populations that have been used to identify QTLs for tomato flavor compounds are summarized in **Table 1**. Tomato has been used as a model crop to demonstrate the IL approach using an interspecific cross between *S. lycopersicum* and a small, green-fruited species *S. pennellii*, a wild relative of *S. lycopersicum*. Some *S. pennellii* accessions and *S. lycopersicum* are sexually compatible despite the adaptation differences including morphology, mating system, flavor, and biotic and abiotic stresses. An IL population of *S. pennellii* composed of 50 lines with overlapping segments in the genetic background of *S. lycopersicum* (cultivar M82) was developed to use for QTL identification and gene cloning (Eshed and Zamir, 1995; Lippman et al., 2007). This population revealed 23 QTL for total soluble solids and 18 for fruit mass (Eshed and Zamir, 1995). Later, a study found Brix9-2-5, an *S. pennellii* QTL that increases the sugar content of tomatoes and is mapped to a gene for a flower- and fruit-specific invertase (*LIN5*) (Fridman et al., 2004). Another study used the *S. pennellii* IL population to evaluate 30 flavor compounds and found 25 loci associated with volatiles and citric acid (Tieman D. M. et al., 2006). A study with *S. pennellii* ILs identified the *SlSAMT* gene encoding a protein that converts salicylic acid to methyl salicylate (Tieman et al., 2010). Using *S. pennellii* ILs, 38 candidate genes with expression correlated with carotenoid accumulation were identified. A transcription factor SlERF6 was determined to play an important role in carotenoid biosynthesis and ripening (Lee et al., 2012). Another study used an IL population derived from a cross between *S. lycopersicum* and *S. habrochaites* (LA1777) and found 30 QTLs affecting 24 volatile compounds including a QTL on chromosome 2 for significantly reduced levels of geranylacetone and 6-methyl-5-hepten-2-one (Mathieu et al., 2009).

An RIL population of 169 lines derived from a cross between *S. lycopersicum* and a red-fruited wild tomato species *S. pimpinellifolium* accession was used to find 102 QTLs for 39 flavor volatiles (Rambla et al., 2017). Another study identified 37 QTLs for 11 fruit quality traits using an interspecific IBL (inbred backcross line) population derived from the cross *S. lycopersicum* cv. Tueza x *S. pimpinellifolium* (LA1589) (Celik et al., 2017). More recently, *S. pimpinellifolium* and IBLs were used to phenotype sugars and organic acids based on SNPs to detect 14 QTLs for sugars and 71 for organic acids (Çolak et al., 2020).

A QTL study using a RIL population made from an interspecific cross between a small-fruited flavorful tomato and a large-fruited variety with poor flavor detected 81 significant associations for 26 traits, including volatiles (Saliba-Colombani et al., 2001). Another study used a RIL population developed from an intraspecific cross between a cherry tomato with good flavor and an inbred with medium flavor and large fruits. They evaluated 38 traits related to organoleptic quality and mapped 130 QTLs (Causse et al., 2002).

Backcross inbred lines (BILs) were also generated by crossing *S. pennellii* and *S. lycopersicum* variety M82; the F_1_ was further backcrossed to M82 for two or more generations followed by selfing for eight generations. It resulted in 446 BILs, each carrying multiple introgressions to allow gene identification controlling wild phenotypes. BILs partitioned the genome into 633 mapping bins with an average of 2.7 introgressions per line. Overall, BILs were shown to enhance mapping resolution of traits introgressed from the wild tomato species (Ofner et al., 2016).

### 3.2 GWAS in flavor

**Figure 9:**
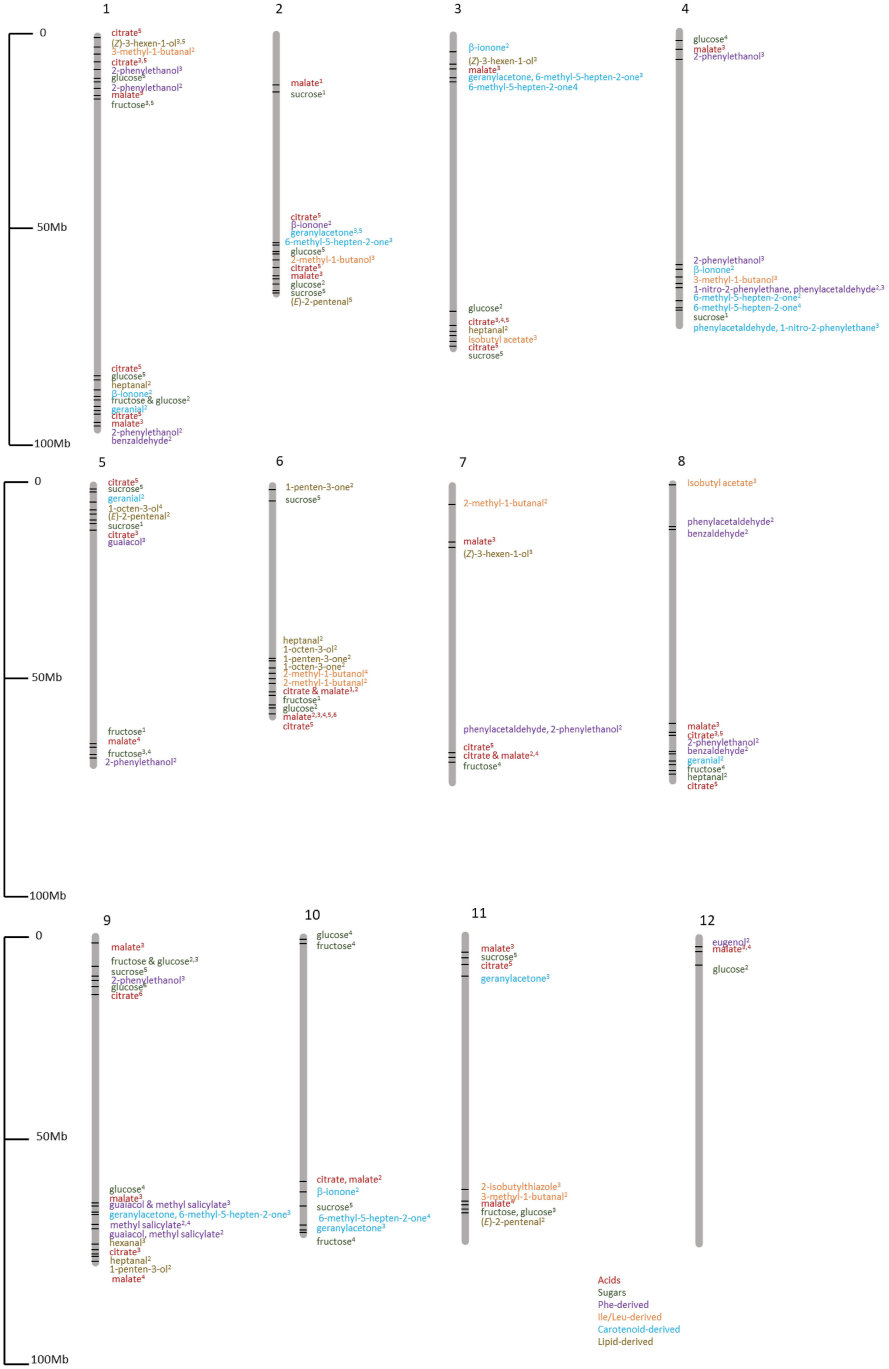
Chromosomal locations of flavor compound GWAS loci identified in multiple GWAS studies. Sauvage et al, 2014 ^1^; Bauchet et al, 2017 ^2^; Tieman et al., 2017 ^3^; Zhao et al, 2019 ^4^ Ye et al., 2019 ^5^; Razifard et al., 2020 ^6^.

Although ILs and QTL mapping have enabled the identification of important loci and genes for flavor compounds, the defined regions are large and contain many candidate genes for each locus. With the developments in whole genome sequencing, significant genetic associations can be found at the genetic level for a desired phenotypic trait. Genome-wide association studies (GWAS) include genotyping a genetically diverse set of individuals considering linkage disequilibrium (LD) between the genetic marker and trait of interest. LD represents the degree to which an allele of an SNP is correlated or inherited with an allele of another SNP in a population. A summary of the GWAS loci identified for tomato flavor compounds in multiple studies is shown in **Table 2** and **Figure 9**. A GWAS based on 96 tomato lines, including Italian and Latin American landraces, wild and modern varieties, identified 20 significant associations for seven fruit traits, namely, β-carotene, fresh weight, trans-lycopene, titratable acidity, ascorbic acid, phenolic compounds, and pH (Ruggieri et al., 2014). Using 163 diverse tomato lines, including *S. lycopersicum*, *S. lycopersicum* var. *cerasiforme*, and *S. pimpinellifolium*, Sauvage et al. (2014) found 44 significant loci for 19 traits, including volatiles, sugars, and acids. This study showed that different metabolic traits have varying genetic controls as a few (such as two loci accounting for 74.3% variation in fruit dehydroascorbate levels) or many (such as five loci for 33.2% accounting for variation in ascorbate levels) genes might be responsible for most phenotypic variation. Another GWAS for 123 cherry tomato lines and 51 large-fruited lines evaluated for 28 volatiles found 125 significant associations; however, this GWAS only used 182 SSR markers, limiting the power of the GWAS (Zhang et al., 2015). A study with a relatively smaller sample of 15 Italian landraces and three commercial F1 hybrids evaluated metabolites belonging to the free amino acid, glycoalkaloid, and phenolic groups (Baldina et al., 2016). The tomato landraces belonged to three fruit-type classes: flattened/ribbed, pear/oxheart, and round/elongate. They found that genetic backgrounds in fruit shape contribute significantly to variation in metabolite levels in tomato.

Zhao et al. (2016) conducted a GWAS using 174 tomato lines composed of *S. lycopersicum* (123 accessions) and *S. lycopersicum* var. *cerasiforme* (51 accessions) and found 58 significant associations for sugars and organic acids. Another study used 300 tomato accessions for 60 primary and secondary metabolites and identified 79 associations for 13 primary and 19 secondary metabolites, including sugars, acids, and VOCs (Bauchet et al., 2017). Tieman et al. (2017) used 398 modern, heirloom, and wild tomato lines for GWAS and found 251 significant associations for 20 traits including sugars, acids, and 15 VOCs. They found that modern tomato varieties have significantly lower amounts of important flavor compounds than heirloom varieties. A meta-analysis of GWAS for tomato flavor based on three GWAS studies included 163 tomato lines from Sauvage et al. (2014), 291 lines from Bauchet et al. (2017), and 402 lines from Tieman et al. (2017), giving a total of 775 tomato lines for 31 flavor traits. They found 37 candidate loci for flavor-related traits including acids, sugars, and VOCs such as 1-penten-3-one, 2-methyl-1-butanol, and 6-methyl-5-hepten-2-one, some of which have been functionally validated (Zhao et al., 2019).

To find answers to the complex biological network for tomato flavor, integrating multiple omics approaches can compensate for the missing information often found when using single omics (Zhu et al., 2019). A systems biology study using genomic, transcriptomic, and metabolomic data from 610 tomato lines comprising 42 wild species lines and 568 red-fruited clade lines (*S. pimpinellifolium*, *S. lycopersicum* var. *cerasiforme*, and *S. lycopersicum*) found that the metabolome is affected by breeding for producer-specific traits such as fruit size. Fruit size genes might not have caused metabolic changes such as primary metabolite content, but the linked genes could have; therefore, precision molecular breeding should be done to reduce the impacts of linkage drag (Zhu et al., 2018). Another GWAS based on 192 tomato lines for six fruit traits including fruit shape, fruit color, pericarp thickness, fruit weight, fruit height, and fruit width found 41 significant loci (Phan et al., 2019). Another study identified 126 significant loci for 92 metabolic traits (flavor and nutrition related) from 302 tomato lines including 171 *S. lycopersicum*, 104 *S. lycopersicum* var. *cerasiforme*, and 27 *S. pimpinellifoliu*m accessions. This study found more significant associations for sugars and organic acids than previously reported by using a large number of diverse accessions (Ye et al., 2019). For example, they identified 17 associations for citric acid whereas up to 4 loci were previously identified (Sauvage et al., 2014; Bauchet et al., 2017).

A study based on GWAS and sweep analyses showed that lower soluble solids are most associated with selection during domestication and the transition to *S. lycopersicum* var. *lycopersicum* (SLL) from *S. lycopersicum* var. *cerasiforme* (SLC) and *S. pimpinellifolium* (SP) using 166 accessions. This population represented SP from near its region of origin in South America and SLC from South America and Mesoamerica, and SLL landraces from Mesoamerica. They also found that fruit size (locule number) and citric acid levels overlap with sweeps in both northward expansion events of SLC, suggesting that some phenotypic changes observed in this expansion may have been driven by selection rather than drift (Razifard et al., 2020). Another study explored genetic variations of five previously cloned tomato flavor genes (*LIN5*, *ALMT9*, *AAT1*, *CXE1*, and *LoxC*) in the same collection, including 166 accessions from South and Central America (Pereira et al., 2021). They showed high genetic variation for these loci, including novel haplotypes not seen in cultivated germplasm. They also investigated functional causative polymorphisms for five loci and, using long-read genome assemblies, resolved a gene duplication at the *LoxC* locus affecting the accumulation of lipid-derived volatiles. The findings were consistent with previous reports of haplotypes associated with improved flavor traits left behind during the transition from wild to cultivated tomatoes.

### 3.3 Pan-genome in flavor

The sequence of the first tomato genome (Heinz 1706) was published in 2012 (Tomato Genome Consortium, 2012); however, this genome did not capture the diversity found in tomato genomes. Although there are many advancements in genetic research, there are still many gaps. A pan-genome comprises all genetic variability of a species and its wild relatives. Gao et al. (2019) constructed a tomato pan-genome using 725 diverse accessions, revealing 4,873 genes absent from the original Heinz 1706 reference genome. They showed gene loss and adverse selection of genes, including flavor genes during domestication and improvement. *TomLoxC* encodes a 13-lipoxygenase previously shown to be associated with the production of C5 and C6 volatiles in tomato. In the orange stage fruit, accessions with both the rare and common *TomLoxC* alleles (heterozygotes) have higher *TomLoxC* expression than those homozygous for either and are resurgent in modern tomatoes. They identified a rare allele in the *LoxC* promoter selected against during domestication and showed that *LoxC* has a role in apocarotenoid volatile production.

Alonge et al. (2020) published high-quality sequences of 14 diverse tomato varieties and identified 238,490 structural variants within these genomes. They identified structural variants that affected flavor volatile accumulation, fruit weight, and the jointless trait in tomato flower pedicels. A recent pan-genome study showed a 24% increase in estimated heritability of 19,353 expression traits and 970 metabolite traits from 332 tomato accessions using a graph genome compared to a single linear reference genome (Zhou et al., 2022).

### 3.4 Transcriptomics in flavor

To better understand the bases of tomato flavor variation, transcriptomics is used to measure differences in gene expression. Multiple resources are available for large-scale transcriptome profiling in tomato (Fernandez-Pozo et al., 2017; Zouine et al., 2017; Shinozaki et al., 2018). The Tomato Expression Atlas (TEA) has been developed as a web tool to store and visualize RNA-seq data from tomato and its closest relative, *S. pimpinellifolium* (Fernandez-Pozo et al., 2017). Another webtool named TomExpress was also developed to browse, visualize, and use RNA-seq data for the tomato research community (Zouine et al., 2017). A global analysis presenting a high-resolution atlas of tomato fruit transcriptome detected 24,660 unique genes in at least one cell or tissue and developmental stage (Shinozaki et al., 2018). This is a great resource to understand the genetic aspects of fleshy fruit development biology in relation to various regulatory mechanisms.

Furthermore, RNA sequencing has proved to be a powerful tool to analyze transcriptome data and to identify genes with differential expression patterns (Maghuly et al., 2021). Expression quantitative trait loci (eQTLs) represent genomic polymorphisms related to gene expression. A study reported 16 candidate genes for fruit ripening based on comparative transcriptome and eQTL analyses (Zhu et al., 2022). Based on the co-expression analysis, they found a candidate gene *SlWD40* with a positive influence on fruit ripening and strongly co-expressed with transcription factors such as *RIN*, *NOR*, *AP2a*, and *SlWRKYs*. Wang et al. (2020) presented a high-quality genome of *S. pimpinellifolium* LA2093 and found 92,000 SV between LA2093 and Heinz 1706. They genotyped these SV using 600 tomato lines and identified alleles under selection during domestication, improvement, and modern breeding. They also used eQTL analysis and detected hotspots for fruit quality traits. One of these hotspots contained a *MYB12* gene, a regulator of flavonoid biosynthesis genes. An AP2/ERF transcription factor gene, *WRI3*, may serve as a master regulator that controls the tissue-specific expression of crucial lipid biosynthetic enzyme genes in tomato fruit epidermis. Another study used cluster analysis and co-expression network construction using transcriptome data and the metabolic contents of 44 VOCs to characterize the mode of inheritance and identified candidate genes by integrating all datasets (Bineau et al., 2022).

### 3.5 Machine learning in flavor

With an increase in tomato breeding efforts towards better flavor and nutritional quality, there is interest in the use of machine learning models to expedite the breeding process (Wang et al., 2022). A study showed that predictive machine learning models can predict consumer preferences based on the metabolomics of tomato and blueberry lines (Colantonio et al., 2022). They found that 42% and 56% of the variance in overall liking was explained by volatiles in tomato and blueberry, respectively. They used 18 statistical and machine learning methods to predict consumer preference and found highest prediction accuracies from the XGBoost, gradient boosting machines, and neural network models. The most predictable traits in tomato were sweetness (0.8), flavor intensity (0.78), and sourness (0.69), whereas in blueberry, it was sourness (0.87) and sweetness (0.75). It was also reported that tomato VOCs including 1-penten-3-one and 2-phenylethanol are important for sweetness perception as estimated by the Gradient Boosting Machines, and (*E*)*-*2-pentenal and 4-carene are important for sweetness enhancement as estimated by the Bayesian model Bayes A. Predictive machine learning models provide an advanced alternative to flavor phenotyping, which is generally expensive and technically challenging. Another study based on 30 populations with the Spanish variety “Moruno” as a reference used partial least square (PLS) models to relate flavor descriptors with sugars, acids, and VOCs (Villena et al., 2023). They found consistent results with the importance of volatiles and sugars to overall flavor and suggested that 6-methyl-5-hepten-2-one is an important volatile to focus on when breeding for flavor in tomato.

### 3.6 Gene editing in flavor

Information provided by the tomato reference genome (100 Tomato Genome Consortium, 2012) and the pan genome (Gao et al., 2019) has facilitated identifying targets for gene editing for several traits in tomatoes. This includes improvement for biotic and abiotic stresses including disease resistance, drought tolerance, fruit yield, quality, and accelerating the domestication of wild varieties (Chandrasekaran et al., 2021; Xia et al., 2021). CRISPR/Cas9 has been utilized to understand the bases of color pigment accumulation in tomato, and to generate fruit with different colors. Pink tomatoes were generated by disrupting the function of tomato *MYB12*, a master regulator of tomato flavonoid biosynthesis, using a CRISPR/Cas9 approach (Yang et al., 2019). Additionally, orange, and yellow tomatoes were generated by knocking out *carotenoid isomerase* (*CRTISO*) and *phytoene synthase 1* (*PSY1*) genes, respectively, using CRISPR/Cas9 (Dahan-Meir et al., 2018). Furthermore, purple tomatoes were generated using targeted insertion of a promoter upstream of anthocyanin biosynthesis gene *SlANT1* in red fruits using CRISPR/Cas9 (Blando et al., 2019).

Apart from fruit color, CRISPR/Cas9 has also been applied to identify volatile biosynthesis genes in tomato flavor. There have been many identified genes that affect tomato flavor volatile synthesis (Pereira et al., 2021; Frick et al., 2022). Using CRISPR/Cas9, it was shown that *FLORAL4* was important for phenylalanine-derived volatile accumulation in tomato fruit (Tikunov et al., 2020). Similarly, Li X. et al. (2020) showed that editing the *Sl-LIP8* gene could significantly alter the levels of three C5 [1-pentanol, (*Z*)*-*2-penten-1-ol, and 1-penten-3-ol] and three C6 [(*Z*)*-*3-hexen-1-ol, (*E*)-2-hexen-1-ol, and hexyl alcohol] VOCs in tomato fruit (Li Q. et al., 2020). Using CRISPR/Cas9, it was shown that NAC transcription factors regulate fruit flavor ester biosynthesis by activating *AAT* expression in tomato, peach, and apple (Cao et al., 2021). Compared to conventional breeding, CRISPR-edited tomatoes could be used to precisely engineer genes in flavor compound biosynthetic genes to improve aroma and flavor of modern commercial tomato varieties.

## 4 Summary and next steps

**Figure 10:**
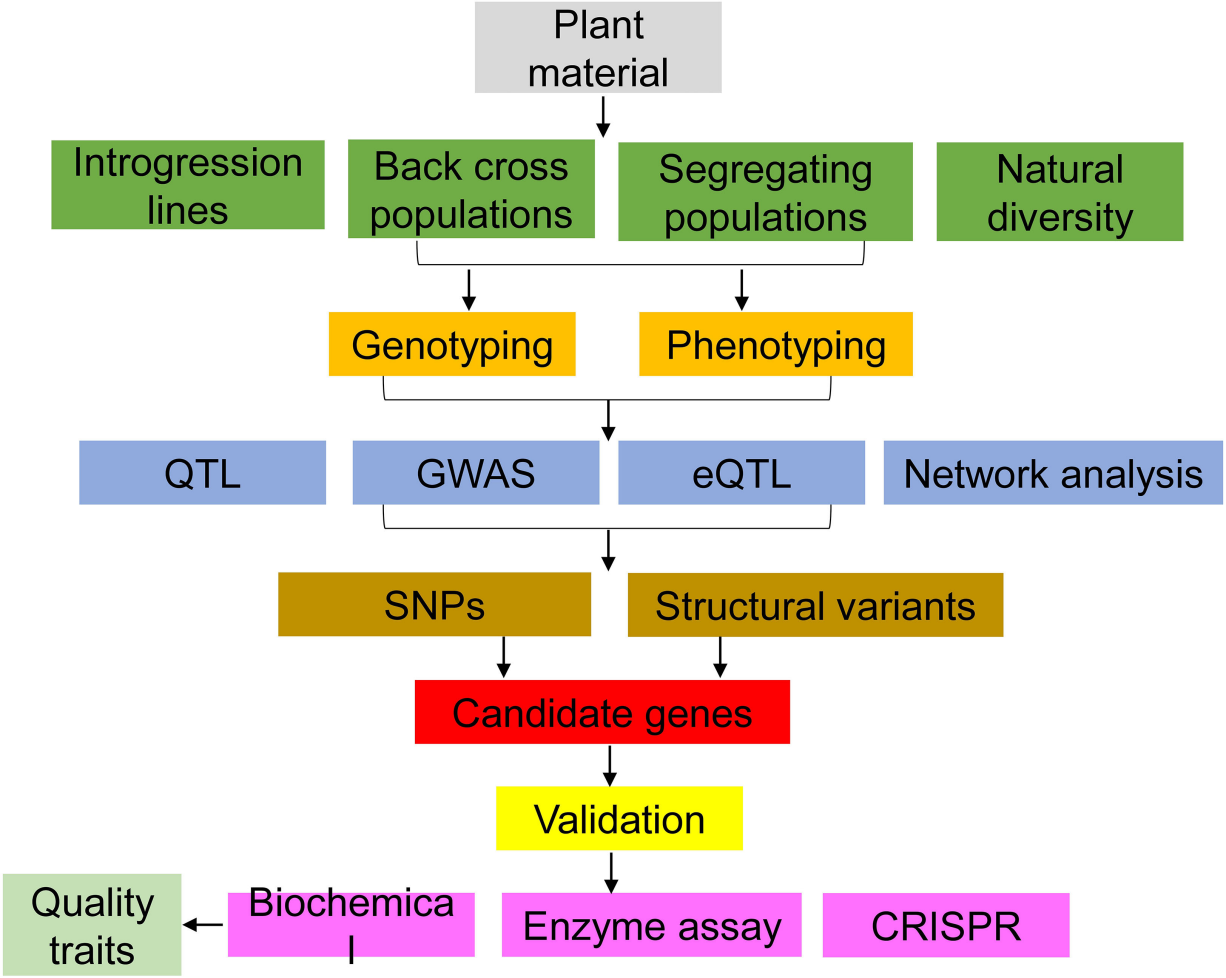
Tools to identify candidate genes for tomato flavor research.

Tomato aroma is a complex mixture of VOCs. Understanding the biosynthetic pathways involved in VOC biosynthesis in tomatoes is a critical step toward developing tomato varieties with desirable flavor traits. Advances in the field of flavor chemistry, genetics, and sensory analysis, including the use of omics, and gene editing, have brought significant progress in understanding the inheritance and metabolic pathways of these tomato aroma volatiles and the associated candidate genes (**Figure 10**). However, there is a long road ahead towards the development of the “perfect tomato”. To do this, additional information and studies on the biosynthetic pathways, regulatory genes, and the chemical modifications that influence VOC emission are critical. While tools applied in these studies continue to evolve, breeders need to prioritize other economic traits like yield and disease resistance while breeding for flavor. Therefore, to avoid linkage drag during breeding, use of advanced tools precisely altering genes of interest should be encouraged in tomato flavor improvement. Such tools, including gene editing, have the capability to reduce the time involved in developing new more flavorful varieties.

## Author contributions

GK and DT conceptualized the study. GK and MA wrote the first draft of the manuscript. All authors contributed to manuscript revision, read, and approved the submitted version.
